# Bariatric Surgery Associates with Nonalcoholic Steatohepatitis/Hepatocellular Carcinoma Amelioration via SPP1 Suppression

**DOI:** 10.3390/metabo13010011

**Published:** 2022-12-21

**Authors:** Shuai Chen, Liming Tang, Adrien Guillot, Hanyang Liu

**Affiliations:** 1 Center of Gastrointestinal Diseases, The Affiliated Changzhou Second People’s Hospital of Nanjing Medical University, Changzhou Second People’s Hospital, Changzhou Medical Center, Nanjing Medical University, Changzhou 213000, China; 2 Department of Hepatology & Gastroenterology (CVK), Charité Universitätsmedizin Berlin, 13353 Berlin, Germany

**Keywords:** diet management, immune environment, key gene exploration, metabolic surgery, NASH-HCC progression

## Abstract

Nonalcoholic steatohepatitis (NASH) is one of the most common chronic liver diseases worldwide and no effective drugs or treatments have been approved for disease management. Recently, bariatric surgery (BS) is considered to be a novel disease-modifying therapy for NASH and liver metabolic diseases, according to clinical follow-up studies. Despite the revealment of physiopathological alterations, underlying mechanisms and key factors remain indeterminate. This study included multiple bulk RNA-sequencing datasets to investigate transcriptome variation in one-year follow-up BS and diet management (Diet) NASH patients’ liver biopsies. Liver functions, fibrosis, and carcinogenesis were predicted in liver samples via hallmark-based function enrichment analysis. Key factors generated from multi-dataset comparison were further assessed with hepatocellular carcinoma (HCC) progression and prognosis. BS leads to active gene expression alterations in NASH liver in comparison to diet management (Diet). Both approaches reduce cell stress and immune response, whereas BS contributes to higher metabolic levels and lower apoptosis levels. The macrophage infiltration, adipose accumulation, and fibroblast activation were revealed to be lower in post-BS NASH livers, further demonstrating positive correlations mutually. Seven key genes (*MNDA*, *ALOX5AP*, *PECAM1*, *SPP1*, *CD86*, *FGF21*, *CSTA*) were screened out as potential macrophage-associated and carcinogenetic factors suppressed by BS. *SPP1* was identified as a crucial factor participating in BS intervened NASH-HCC progression. This study determined that BS exerts potentially superior protective functions in NASH livers compared to diet management. SPP1 may serve as a novel factor to study the functionalities of BS on NASH patients.

## 1. Introduction

Gradually increasing with the prevalence of obesity, non-alcoholic fatty liver disease (NAFLD) has become the heaviest burden of chronic liver disease worldwide [1,2]. The global prevalence of NAFLD is 25.24%, the presence of non-alcoholic steatohepatitis (NASH) was found in approximately 59.10% of patients with biopsied NAFLD, and annual cumulative hepatocellular carcinoma (HCC) incidence of NASH-related cirrhosis was found to be 2.6% [3]. Continuous fibrosis and cirrhosis act as leading causes NASH-derived HCC [4]. Despite advances in disease research, no effective drugs have been approved as therapies for NASH [5].

Fat deposition plays a crucial role in the pathophysiology of NAFLD. It has been reported that morbidly obese patients (body mass index > 40 kg/m^2^) represent more than 90% of patients with NAFLD, 30% of whom are NASH, while up to 5–10% of subjects progress to cirrhosis [6,7]. Current evidence suggests that weight loss by 3–5% and 5–7% remises hepatic steatosis and inflammation, respectively. In addition, weight loss by more than 10% attenuates liver fibrosis [8]. Thus, weight loss has been considered as one of the most definitive treatments for NASH [9]. Currently, weight loss is executed by lifestyle alterations, (including diet management and extra exercise), metabolic medicine, and Bariatric Surgery (BS) [10,11]. Although the primary treatment for NAFLD continues to be weight loss through exercise and lifestyle changes, only a minority of individuals achieve sustained weight loss. Moreover, pharmacological weight loss strategies have limited effectiveness. BS, on the other hand, can provide long-term weight loss and improvement in obesity-related diseases. BS has been demonstrated to have an important and beneficial role in the management of obesity [12]. BS not only results in significant weight loss but also significantly improves insulin sensitivity and reduces type 2 diabetes [13,14]. Due to the association between metabolic syndrome and NAFLD, BS has the potential to produce lasting and meaningful improvements in liver-related metabolic disease [15]. Currently, although malabsorption and hormone alteration are regarded as critical mechanisms in post-BS patients, it remains unclear about the effect of molecular functions on weight loss and multi-organ metabolic improvement [16]. Recent clinical studies have found a significant reduction in the risk of tumor development in patients after BS [17]. Then, the mechanism by which this occurs has not been elucidated.

Accordingly, we propose a hypothesis that BS can potentially favor NAFLD/NASH patients, not only by attenuating hepatic steatosis but also reducing hepatic carcinogenesis. This study investigated potential effects on liver recovery, therefore determining steatosis, fibrosis, and carcinogenesis in post-BS and Diet NASH livers, via transcriptome analysis. Furthermore, key immune cells and genes were identified, and underlying biological interactions were predicted.

## 2. Materials and Methods

### 2.1. Data Source and Analysis

The datasets were obtained from the Gene Expression Omnibus (GEO) database, and characterized information is listed in supplementary Table S1. To consolidate NASH-BS samples, the GSE106737 and GSE83452 were merged using the R package inSilicoMerging [18], and batch effects were remised using the method of Johnson WE et al. [19] (supplementary Figure S5). Differentially expressed genes (DEGs) between groups were sorted out by limma package [20] with log (fold change, FC) > 1.5 and *p* < 0.05. Overlapping analyses among multiple gene clusters were carried out by Venn diagraming [21]. SPP1 expressions in situ (normal tissue vs. tumor tissue) were generated from Human Protein Atlas (HPA) https://www.proteinatlas.org/ (accessed on 8 April 2022).

### 2.2. Function Enrichment Analysis and Protein-Protein Interaction (PPI) Network

Function enrichment analysis was conducted with the Gene set enrichment analysis (GSEA) [22], according to the latest database of Kyoto Encyclopedia of Genes and Genomes (KEGG), Gene ontology (GO), Hallmarks and cancer modules. A minimum gene set of 5 and a maximum gene set of 5000 were set, and *p* < 0.05 and false discovery rate (FDR) < 0.25 were considered as significant. PPI networks were generated by STRING, and further organized by Cytoscape software (version 8.3, Institute of Systems Biology, Seattle, USA). In addition, hub clusters and gene were classified with the MCODE plugin (k-core = 2) [23].

### 2.3. Gene Profiling in Liver Hepatic Carcinoma (LIHC)

The Gene Expression Profiling Interactive Analysis (GEPIA) [24] was applied to investigate LIHC and normal liver tissue respectively based on gene profiling of TCGA and the Genotype-Tissue Expression (GTEx) [25]. Expression levels, overall survival (OS), disease-specific survival (DSS), progression-free survival (PFS), and disease-free survival (DFS) were analyzed associated with candidate genes. Survival time was analyzed and displayed using the Kaplan–Meier plotter. The mean follow-up time was above 10 years, and Cox *p*-value was analyzed. The gene mutation landscape was analyzed to reveal related oncogene mutation by ComplexHeatmap R package and plotted by the oncoprint [26].

### 2.4. Cell Enrichment Analysis

xCell was used to assess the distribution of 64 immune and stromal cell species in tissues, based on gene expression profile [27]. Therefore, raw enrichment scores and immune scores emerged. The Tumor IMmune Estimation Resource (TIMER) database was introduced to assess the main immune cell infiltration and related cancer outcome, based on the Cancer Genome Atlas (TCGA) database [28].

### 2.5. Weighted Gene Co-Expression Network Analysis (WGCNA)

A co-expression network was constructed using the WGCNA R package [29] in post-BS subgrouping gene expression profile. The correlation analysis implemented in the WGCNA package is based on the Pearson method, and specific parameters (minModuleSize = 30, reassignThreshold = 0, and mergeCutHeight = 0.25) were used to run the program. Then, the clusters of co-expressed genes were displayed as modules labeled in multiple colors, with indications of correlation coefficient values and significance (*p* values).

### 2.6. Mouse Models

Animal experiments were approved by the Laboratory Animal Welfare Ethics Committee of Nanjing Medical University. Male C57BL/6J mice (4–6 weeks old) were obtained from the animal core facility of Nanjing Medical University (Jiangsu, China). The mice were housed in specific pathogen-free units of the Animal Center at the Affiliated Changzhou No. 2 People’s Hospital of Nanjing Medical University (with a 12-hour day-night cycle, 23 ± 1 °C, 60–70% humidity) and fed a 60% high-fat diet (HFD; D12492, Research Diets, Inc., New Brunswick, NJ, USA) for 16 weeks. Living status and weights were measured and recorded every 2 days. Mice of the BS model went through BS following the Sleeve gastrectomy surgical procedure [30]. Mice of the diet management model were fed normal diet. Mouse livers were harvested at the 8-week time point. Animal experiments were approved by the Animal Welfare Committee of the affiliated Changzhou No. 2 People’s hospital of Nanjing medical university [Approval No. (2020) KYO 045-05].

### 2.7. RNA Quantification

Livers were harvested from mouse models (4 from BS; 4 from Diet). For each liver, four tissue samples were collected from separated locations and then smashed with a Homogenizer (QIAGEN, Hilden, Germany). Total RNA was extracted from tissue homogenate using TRIzol**^®^** reagent (Invitrogen; Thermo Fisher Scientific, Waltham, MA, USA). Reverse transcription was conducted with a PrimeScript™ RT Reagent Kit (Takara Biotechnology Co., Ltd., Tokyo, Japan), and real-time quantification was subsequently performed using a SYBR**^®^** Premix Ex Taq Kit (Takara Biotechnology Co., Tokyo, Japan). The relative RNA expression levels were calculated using the −ΔΔCt method. Mouse primers included in this study are listed in supplementary Figure S1.

### 2.8. Statistical Analysis

SPSS 26.0 (SPSS statistics, IBM, New York, NY, USA) was used for general statistical analysis. GraphPad Prism 9.0 software (GraphPad Software, Inc., San Diego, CA, USA) and R Studio were used to generate plots. Significant differences were identified using one-way ANOVA with the Bonferroni post hoc test. Pearson correlation analysis was performed to calculate the correlation coefficients. Data are presented as the mean ± SEM values. “*p* < 0.05” commonly indicates a statistically significant difference.

## 3. Results

This study aims to reveal evidence about molecular functions and prognostic indications of BS and diet management (Diet) on NASH livers. Through bioinformatic analysis on specific RNA-seq datasets, BS is demonstrated as a superior suppressor of cell stress and immune response in human NAFLD/NASH livers. In addition, *SPP1* is screened out and regarded as a decisive factor participating in BS-intervened NASH-HCC progression. Furthermore, we verify the gene expression in liver samples derived from BS + HFD mouse models. 

### 3.1. DEGs Mining and Function Enrichment

**Figure 1:**
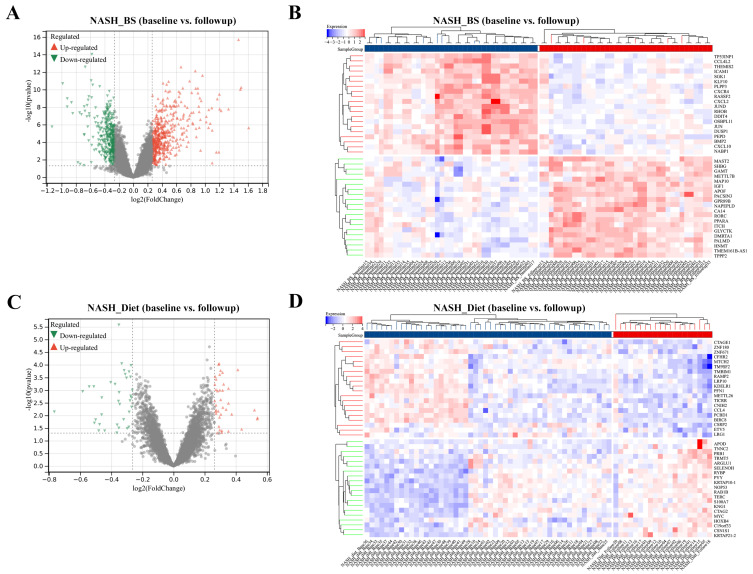
Identification of DEGs. DEGs were screened out from NASH_BS (**A**) and NASH_Diet (**C**) subgroups. The top 20 total significant upregulated and downregulated DEGs were mapped with gene profiles from NASH_BS (**B**) and NASH_Diet (**D**) subgroups.

In the BS group, 322 up-regulated DEGs and 465 down-regulated DEGs were screened out (Figure 1A) and the top 20 DEGs were displayed in a matrix profile (Figure 1B). In the Diet group, 36 up-regulated DEGs and 38 down-regulated DEGs were screened out (Figure 1C) and the top 20 DEGs were displayed in a matrix profile (Figure 1D). Based on DEGs, functional interactions were assessed by PPI analysis. With the MCODE method, highly interconnected clusters were identified (in red or green) and classified within and BS group and Diet group (Supplementary Figure S1). From each cluster, hub genes were marked in yellow, potentially exerting key functions. Results indicate that BS leads to more active influences and transcriptome alterations in NASH liver than Diet.

**Figure 2:**
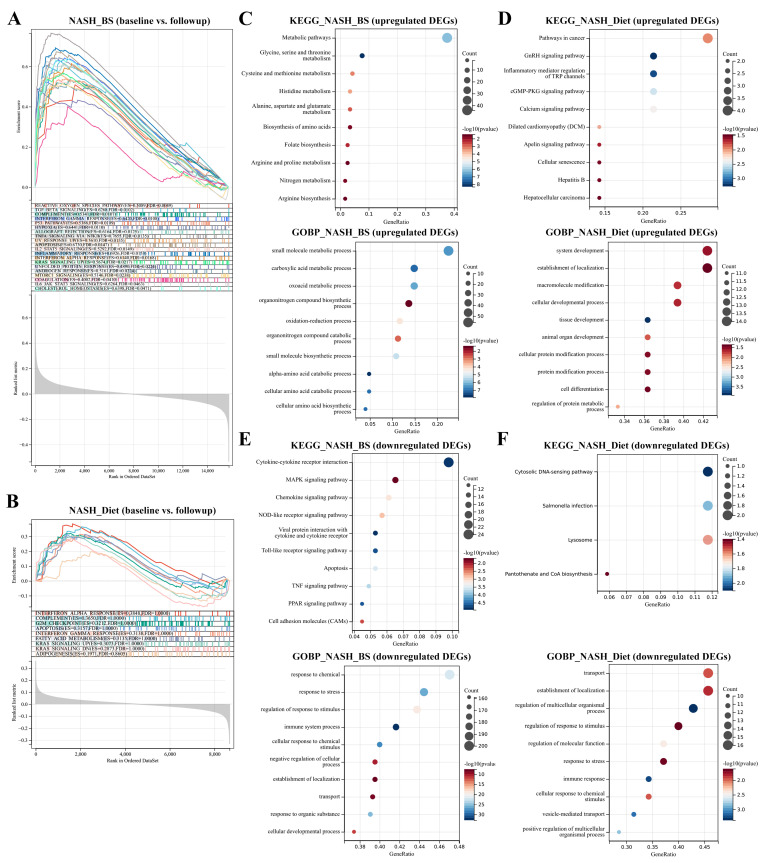
Function enrichment. Hallmark enrichment analyses were conducted in the NASH_BS and NASH_Diet subgroups. (**A**) 20 significant hallmark terms were shown with enrichment scores (ES) in NASH_BS subgroup (FDR adjusted *p* < 0.05). (**B**) 9 top-ranked (but not significant) hallmark terms were shown in the NASH_Diet subgroup (FDR adjusted *p* > 0.05). KEGG and GO enrichment analyses were conducted in NASH_BS and NASH_Diet subgroups. Top significant KEGG and GO terms from upregulated and downregulated gene clusters were respectively displayed (**C**–**F**) (*p* < 0.05).

To further understand hallmark distribution in cohorts, the GSEA was introduced and all significant hallmarks were sorted out. In the BS group, a total of 20 hallmarks, notably including TNF-α signaling via NF-κB, inflammatory response, apoptosis, p53 pathway, Kras pathway, cholesterol homeostasis, and TGF-β pathway, were enriched in baseline samples (Figure 2A). Contrastively, there were no significant hallmarks enriched in the Diet group (Figure 2B). Simultaneously, KEGG and GO-Biological process (BP) were used to predict innate functions and BPs. Generally, in the BS group, metabolism-related processes are enriched in upregulated genes (Figure 2C) and cell stress-related processes are enriched in downregulated genes (Figure 2E); in the Diet group, cancer-, inflammation- and tissue-related processes in upregulated genes (Figure 2D) and cell stress- and immune-related processes in downregulated genes (Figure 2F). Results indicate that both BS and Diet interventions can attenuate cell stress. Specifically, BS favors multiple metabolic processes and attenuates inflammation and cell death. Interestingly, Diet appears to activate tissue development as well as cancer-related processes.

### 3.2. BS Attenuates Risks of Inflammation, Steatosis, and Fibrogenesis in NASH Liver, Superior to Diet

**Figure 3:**
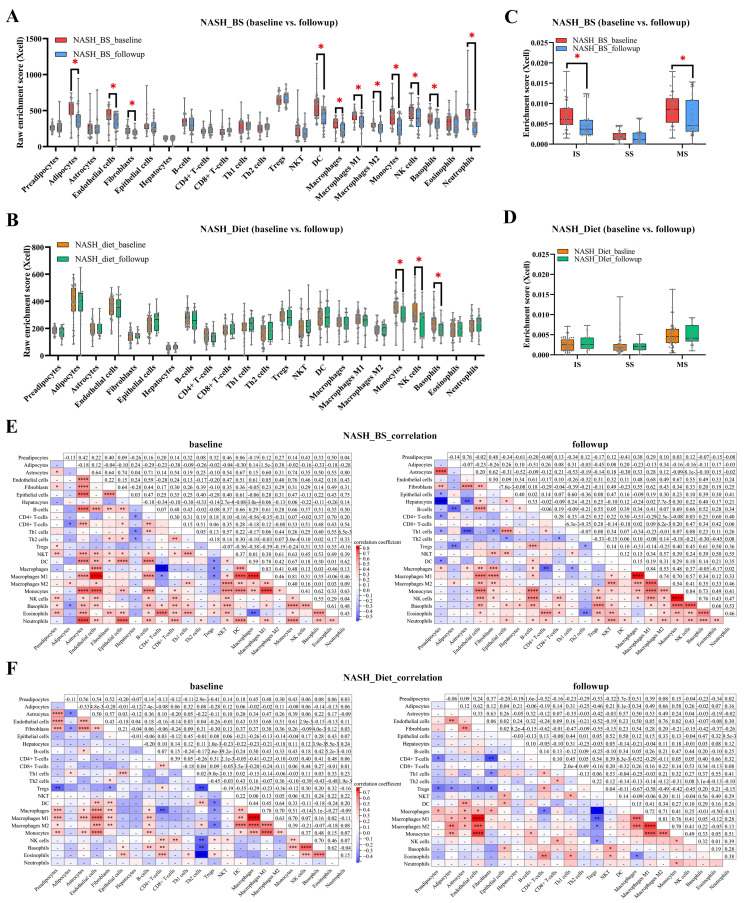
Cell type enrichment. Cell type enrichment levels of in total 7 parenchyma and 16 non-parenchyma cell types were displayed as NASH_BS (**A**) and NASH_Diet (**B**) subgroups. Immune scores were displayed as NASH_BS (**C**) and NASH_Diet (**D**) subgroups (*: *p* < 0.05). Pearson’s correlation analyses were carried out mutually among 23 cell populations in NASH_BS (**E**) and NASH_Diet (**F**) subgroups (*: *p* < 0.1, **: *p* < 0.01, ***: *p* < 0.001, ****: *p* < 0.0001).

Cell type enrichment and immune infiltration were evaluated to exhibit the landscape of major none-and parenchyma cells by the Xcell analysis method. In the BS group (baseline vs. follow-up), enrichment levels of adipose markers (annotated as “adipocytes”), endothelial cells, fibroblasts, dendritic cells (DCs), macrophages (M1- and M2-), monocytes, nature killer (NK) cells, basophils, and neutrophils went down significantly (Figure 3A). In contrast, enrichment levels of monocytes, NK cells, and basophils went down significantly in the Diet group (Figure 3B). Thereby, immune score (IS), stromal score (SS), and microenvironment score (MS) were assessed with the Xcell method. IS and MS were found significantly reduced in the BS group (baseline vs. follow-up) (Figure 3C). In contrast, no significant alterations were found in the Diet group (Figure 3D). Furthermore, to reveal correlative intercellular functions, Pearson’s correlation was applied to cell enrichments. From parenchyma cell populations, adipocytes and fibroblasts were investigated to indicate steatosis and fibrogenesis. Respectively, in the BS-baseline subgroup (Figure 3E), the adipose markers showed a positive correlation with M2 macrophages, and negative correlations with CD8^+^ T cells and basophils (|coef| > 0.3, *p* < 0.05). Fibroblasts showed positive correlations with epithelial cells, B cells, Th2 cells, NK T cells, DCs and eosinophils (|coef| > 0.3, *p* < 0.05). In the BS-follow-up subgroup, adipocytes showed negative correlations with B cells and Tregs (|coef| > 0.3, *p* < 0.05). Fibroblasts showed positive correlations with macrophages (total, M1 and M2), monocytes, NK cells and NK T cells, and negative correlations with Th1 cells (|coef| > 0.3, *p* < 0.05). Accordingly, in the Diet-baseline subgroup, adipocytes showed a positive correlation with Th1 cells, and negative correlations with astrocytes and fibroblasts (|coef| > 0.3, *p* < 0.05). Fibroblasts showed positive correlations with Th2 cells, DCs, and macrophages (total, M1, and M2), and negative correlations with Tregs (|coef| > 0.3, *p* < 0.05). In the Diet-follow-up subgroup (Figure 3F), adipocytes showed positive correlations with endothelial cells, macrophages (M1 and M2) and monocytes (|coef| > 0.3, *p* < 0.05). Fibroblasts showed a positive correlation with macrophages, monocytes, and negative correlations with CD4^+^ T cells, Th1 cells, and Tregs (|coef| > 0.3, *p* < 0.05). Variable correlation results suggest that BS and Diet lead to differential effects on steatosis and fibrogenesis. Particularly, macrophages potentially play vibrant roles in post-BS status, eventually resulting in risk attenuation of steatosis and fibrogenesis.

### 3.3. Key Factor Identification in Post-BS NASH Livers

**Figure 4:**
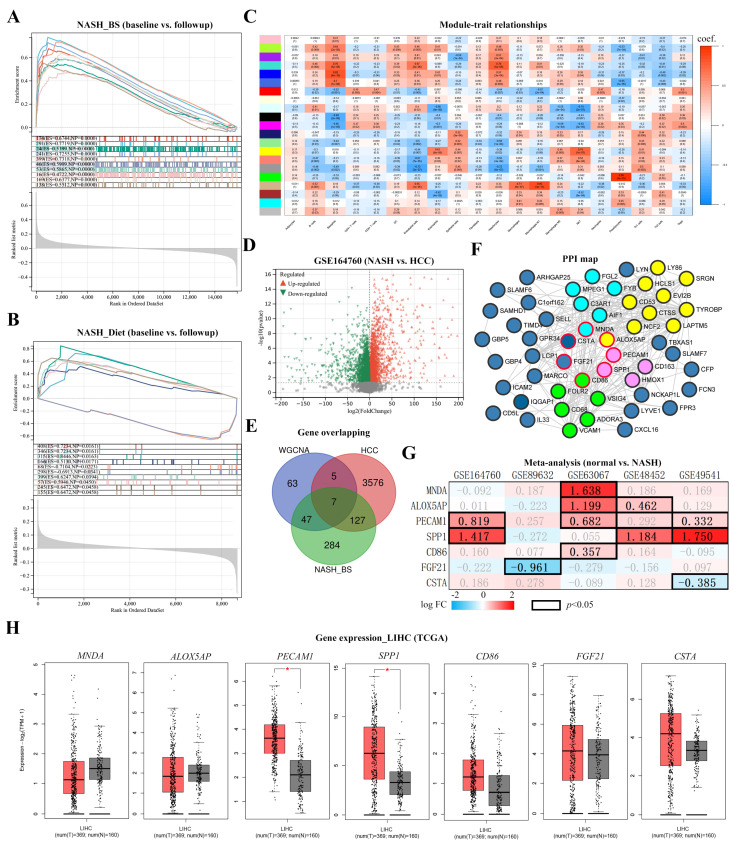
Identification of cancer-associated key genes in NASH_BS livers. Enriched cancer modules were assessed and displayed as (**A**) NASH_BS and (**B**) NASH_Diet subgroups. (**C**) High macrophage-associated gene modules in the NASH_BS subgroup were selected according to WGCNA. Coef [p (scientific notation: e.g., 2e-05 = 2 × 10^−5^)] values were indicated in each block. (**D**) DEGs were screened out from GSE164760 (NASH vs. NASH-derived HCC). (**E**) Overlapping genes were generated from downregulated genes in the NASH_BS subgroup, upregulated genes in the NASH-HCC group, and macrophage associated WGCNA module. (**F**) The PPI network was generated with 54 overlapping genes from downregulated genes in the NASH_BS subgroup and the macrophage-associated WGCNA module. Hub gene clusters were colored light blue, purple, and green. And cancer associated genes were boarded in red. (**G**) Fold changes (FC) of gene (*MNDA*, *ALOX5AP*, *PECAM1*, *SPP1*, *CD86*, *FGF21* and *CSTA*) expression were meta-analyzed in 5 datasets. FC values with statistical significance (*p* < 0.05) were highlighted. (**H**) Gene (*MNDA*, *ALOX5AP*, *PECAM1*, *SPP1*, *CD86*, *FGF21* and *CSTA*) expression in healthy and tumor tissues were displayed based on TCGA database (*: *p* < 0.01).

Based on the GSEA, cancer modules (CM) were used to evaluate cancer conditions in datasets. In the BS group, all significant CMs were enriched in baseline samples (*p* < 0.05) and the top 10 CMs are listed in Figure 4A. Contrastively, in the Diet group, there were 2 of the top 10 significant CMs enriched in follow-up samples (Figure 4B). Results suggest that the BS may exert anti-cancer effects, superior to the Diet. To investigate relationships between cell infiltrations and cancer outcomes, survival analysis was generated by the Kaplan–Meier method based on the TCGA database, respectively with infiltration levels of monocytes, macrophages (total, M1 and M2), DCs, NK cells, endothelial cells, and neutrophils. Compared with high infiltration levels, low infiltration levels of macrophages and neutrophils contribute to a longer overall survival time (*p* < 0.05). Interestingly, infiltration levels of macrophages and neutrophils were significantly reduced in NASH liver after the BS (Supplementary Figure S2).

WGCNA was carried out to determine macrophage correlated gene modules (details regarding scale independence, the mean connectivity, cluster Dendrogram, eigengene adjacency heatmap are displayed in supplementary Figure S3). The module-trait relationships are displayed in a heatmap (Figure 4C), from which the “MEtan” module (containing 142 genes) was selected with superior correlation (coef = 0.71) and significance (*p* < 0.05). The NASH-HCC bulk RNA-sequencing dataset (GSE164760) was obtained and 3487 upregulated and 2606 downregulated DEGs were screened out (NASH vs. NASH-derived HCC, *p* < 0.05) (Figure 4D). The match-up analysis was conducted from the “MEtan” module by WGCNA, upregulated DEGs in NASH-HCC dataset, and downregulated DEGs in the BS group, generating 7 overlapping genes (*MNDA*, *ALOX5AP*, *PECAM1*, *SPP1*, *CD86*, *FGF21*, *CSTA*) (Figure 4E). 54 overlapping genes were selected from the “MEtan” module by WGCNA and downregulated DEGs in the BS group were mapped with PPI. Therefore, hub clusters (marked in light blue, light purple, and light green) were identified by the MCODE method. Moreover, 7 cancer-related genes were boarded in red (Figure 4F).

**Figure 5:**
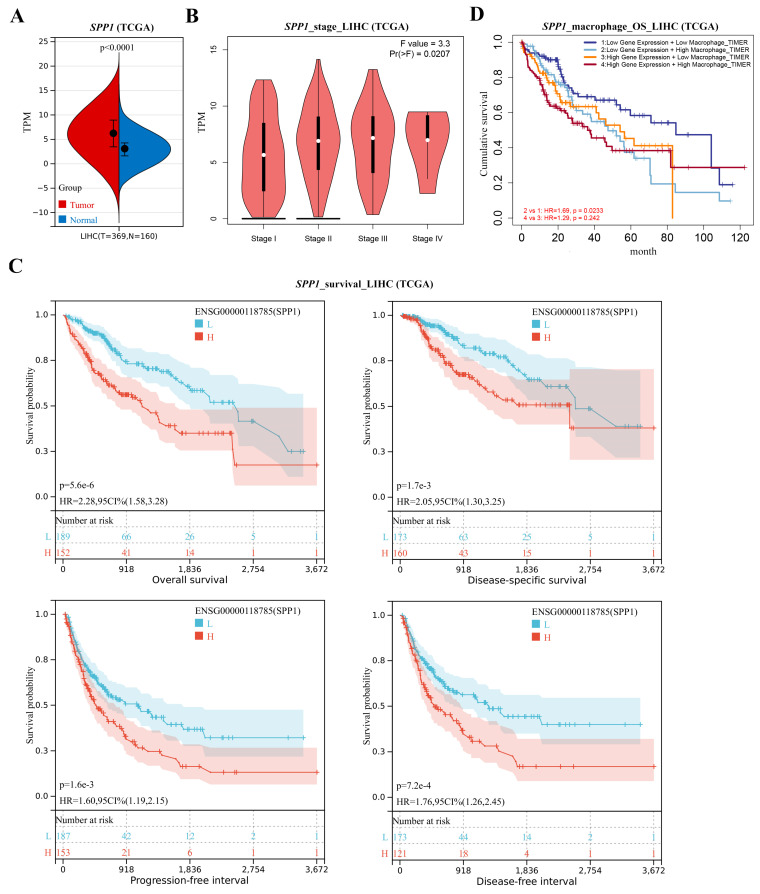
Annotation of *SPP1* in LIHC/HCC. (**A**) *SPP1* expression was compared in LIHC (TCGA) dataset. (**B**) The correlation of *SPP1* expression and tumor stages was analyzed in LIHC (TCGA) dataset. (**C**) Correlation of *SPP1* expression and survival time (OS, DSS, PFS, and DFS) was analyzed in LIHC (TCGA) dataset. *p* value is displayed in scientific notation (e.g., 1.6e-3 = 1.6 × 10^−3^). (**D**) Correlation of OS, *SPP1* expression, and macrophage infiltration levels was analyzed in the LIHC (TCGA) dataset.

Gene expression levels of 7 selected genes were confirmed in liver hepatocellular carcinoma (LIHC) or HCC datasets based on NASH (GSE164760, GSE89632, GSE63067, GSE48452) and liver fibrosis (GSE49541) datasets and TCGA. *SPP1* showed the highest fold change increase in NASH and advanced fibrosis livers (*p* < 0.05) (Figure 4G). *SPP1* and *PECAM1* were found significantly upregulated in tumor tissues (Figure 4H). The gene mutation landscape was analyzed according to the *SPP1* expression. Leading mutations were found on *TP53* (38.9%) and *CNNB1* (32.8%), which suggests a high risk of carcinogenesis and malignancy (Supplementary Figure S4A). The *SPP1* expression was revealed as a relatively high level in tumor tissues (*p* < 0.05) (Figure 5A) and a positive correlation with tumor staging (*p* < 0.05) (Figure 5B). In addition, the relatively low *SPP1* expression dramatically extended survival periods (OS, DSS, PFS, and DFS) for LIHC/HCC patients (*p* < 0.05) (Figure 5C). SPP1-related immune infiltrations in LIHC were analyzed by TIMER. Positive correlations were determined between the *SPP1* expression and infiltrations of B cells, CD4^+^ T cells, macrophages, neutrophils, and DC (*p* < 0.05) (Supplementary Figure S4B). Filtering with differential immune infiltrations in post-BS NASH liver, the macrophages, neutrophils, and DC was regarded as significant cell populations. In the low-SPP1 subgroup, the co-occurrence with low macrophage infiltration led to even longer OS periods (*p* < 0.05) (Figure 5D). OS periods were also analyzed with co-occurrent infiltrations of neutrophils and DCs, which did not show significant differences in low-SPP1 subgroups (supplementary Figure S4C,D). Results indicate that high *SPP1* expression contributes to carcinogenesis and malignancy in NASH liver. Thus, by the co-attenuation of the *SPP1* expression and macrophage infiltration, BS potentially acts as a novel protective intervention for LIHC/HCC prognosis.

### 3.4. Spp1 Expression Was Attenuated after BS and Associated with NASH/HCC Biomarkers

**Figure 6:**
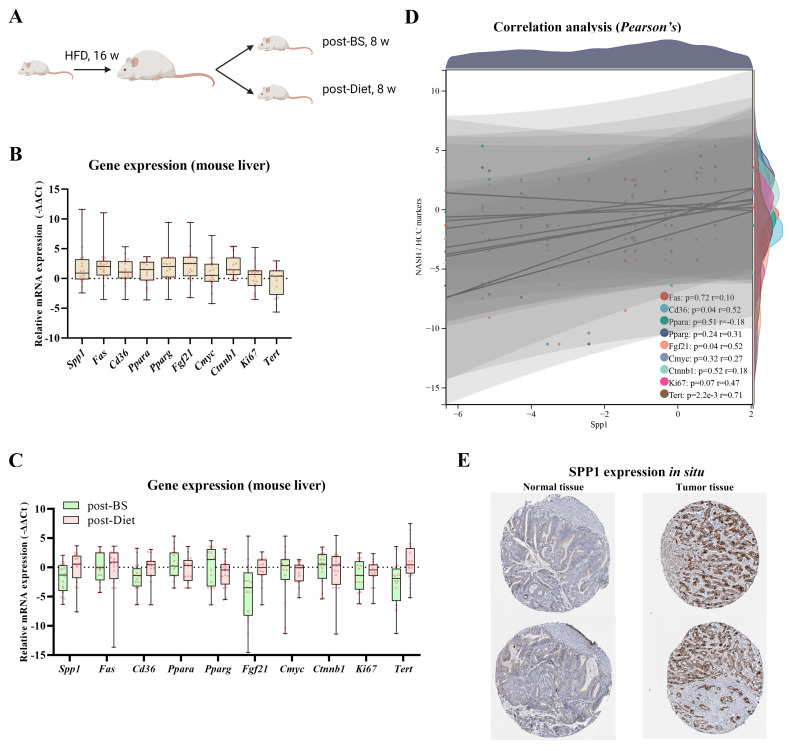
*SPP1* expression in mouse models and patients. (**A**) Post-BS and -Diet on obese mouse models were performed. Relative gene expressions of Spp1 and 9 NASH/HCC biomarkers (*Fas*, *Cd36*, *Ppara*, *Pparg*, *Fgf21*, *Cmyc*, *Ctnnb1*, *Ki67,* and *Tert*) were displayed in obese (**B**), Post-BS, and -Diet mouse livers (**C**). (**D**) Correlation analyses between *Spp1* and 9 NASH/HCC biomarkers (*Fas*, *Cd36*, *Ppara*, *Pparg*, *Fgf21*, *Cmyc*, *Ctnnb1*, *Ki67,* and *Tert*) were respectively conducted following the Pearson’s method. (**E**) SPP1 expressions in situ (normal tissue vs. tumor tissue) were generated from the HPA database.

Post-BS and -Diet on obese mouse models were established following a 24-week procedure (Figure 6A). Gene expressions of *Spp1* and 9 NASH/HCC biomarkers (*Fas*, *Cd36*, *Ppara*, *Pparg*, *Fgf21*, *Cmyc*, *Ctnnb1*, *Ki67*, and *Tert*) were quantified from obese, post-BS and -Diet mouse livers. All ten genes were overexpressed in mouse liver tissue after 16-week HFD feeding compared to normal mouse liver tissue, indicating the occurrence of hepatic steatosis and fibrogenesis (Figure 6B). Compared to un-intervened obese mouse livers, *Spp1*, *Cd36*, *Fgf21*, *Ki67*, and *Tert* tended to be suppressed in post-BS obese mouse livers. And only *Pparg* expression tended to be suppressed post-Diet mouse livers (Figure 6C). In post-BS obese mouse livers, *Spp1* expression revealed a significantly positive correlation with *Cd36*, *Fgf21*, and *Tert* (R > 0.3, *p* < 0.05) (Figure 6D). *SPP1* expressions in situ were obtained from the Human Protein Atlas (HPA) and displayed in immunohistochemistry images. In contrast to normal liver tissue, *SPP1* showed a dramatic overexpression in HCC tissue (Figure 6E). Results revealed that *SPP1*, which might act as a NASH/HCC indicator, was attenuated after the BS intervention.

## 4. Discussion

Commonly caused by constant obesity, NASH is a complex of fat deposition, fatty acid metabolism, and inflammatory modulation [31,32]. The fat overload in liver leads to disease progression by developing steatosis, fibrosis, cirrhosis, and HCC [33,34]. BS has been regarded as the influential management for progressive obesity that contributes to efficient weight loss and the remission of obesity-associated metabolic diseases, such as NASH [35]. In recent years, several clinical studies tracked multiple patient cohorts and demonstrated the efficacy of BS in the treatment of NAFLD [36]. Notably, attenuation of steatosis, inflammation, and fibrosis were discovered in post-BS patients in 1 to 5 years [37]. Due to the invasive intervention, BS is not recommended by the American Association for the Study of Liver Diseases (AASLD) for special NASH treatment. However, BS should be considered as an alternative for obese NASH patients (BMI ≥ 35 kg/m^2^) to improve liver functions and disease progression [38,39]. From the long-term view, results support that protection against NASH and liver fibrosis by BS is superior to diet management or pharmacological interventions [40,41,42,43,44]. Gradually, physiological mechanisms are revealed, mainly referring to gut hormones, bile acid homeostasis, and adipose tissue dysfunction. However, due to the limitation of follow-up, it is difficult to record the incidence of HCC, thus few studies have either inspected it or provided evidence.

In this study, transcriptome alterations were investigated and compared in post-Diet and -BS NASH liver samples (with one-year follow-up). From each subgroup, significant DEGs were screened out. Through function and cell type enrichment analysis, viable indexes including disease hallmarks, biological processes, signaling pathways, and immune cell infiltration were evaluated. Similar to reported conclusions, lipid deposition, cell stress, and inflammatory responses tend to decrease after both obesity managements (BS and Diet). Notably, metabolic improvement, remission of adipose accumulation, and fibroblast activation and inflammatory response were uncovered as more active in post-BS NASH livers, suggesting that BS potentially leads to liver recovery and fibrosis attenuation. In NAFLD, several emerging inflammatory mechanisms have been uncovered, including profound macrophage heterogeneity [45]. Interestingly, adipose accumulation and fibroblast activation revealed positive correlations with macrophage infiltration in post-BS NASH livers, which indicated the crucial roles played by macrophages. However, despite the multifarious cell type evaluation provided by Xcell, the bias of cell classification appears to be a nonnegligible limitation. Moreover, macrophage heterogeneity and M1/M2 classification are oversimplified. To figure out further interaction among cell clusters, single-cell RNAseq analysis may be necessary. Since liver fibrosis could eventually contribute to carcinogenesis, another dataset was introduced in this study, containing liver samples with NASH and NASH-derived HCC. By comparing downregulated and macrophage-associated gene clusters in post-BS NASH liver with the upregulated gene cluster in NASH-HCC liver samples, 7 genes were screened out, among which *SPP1* was ultimately identified with significant relevance of progression and prognosis in HCC patients. In the end, *SPP1* expressions were verified in both mouse models and patients.

Secreted phosphoprotein 1 (encoded by *SPP1*), also known as osteopontin (OPN), is proven secretive. Exploiting from the Human protein Atlas (HPA), SPP1 is inactive in the majority of healthy liver cells under healthy conditions, except Kupffer cells and cholangiocytes. According to GO annotations, SPP1 participates in extracellular interaction, fibroblast growth, and immune regulation. Studies have reported that SPP1 participates in inflammation, liver fibrosis, and HCC, therefore taking center roles in chronic liver diseases [46,47,48]. In addition, this study revealed that the co-occurrence of low *SPP1* expression and low macrophage infiltration led to dramatically longer OS time for HCC patients. In summary, results from this study validated clinical conclusions from transcriptome levels, whereby the identification of SPP1 provides a breakthrough point to explore deep mechanisms in BS-NASH aspects. 

Nonetheless, it is still challenging to link surgical interventions to exact molecular functions. Animal models were applied in this study. However, we were only able to conduct a correlation analysis between *SPP1* expression and disease markers, which could be a robust limitation. To further improve evidence, more in situ approaches can be used, such as the multiplex immunofluorescent staining method [49]. Critical limitations usually lead to abortion of further studies, such as lack of suitable in vivo models to investigate both BS and NASH-derived HCC [50,51]. Moreover, it seems unsolvable to mimic post-surgery status with in vitro models, which is mandatory for molecular validation. Potentially, single-cell and spatial transcriptome analysis technics may be beneficial to underlying the post-BS liver niche, under the circumstances that in vitro experiments are still far [52,53]. Conclusively, according to the bioinformatic and biological investigation, BS exerts potentially superior effects on ameliorating progressions of NASH patients’ in comparison to the diet management. Furthermore, BS significantly depressed SPP1 expression in NASH livers, which can potentially reverse poor prognosis in NASH-derived HCC patients. Therefore, SPP1 may serve as a novel functional factor to study the functions and mechanisms of BS on NASH patients.

## 5. Conclusions

According to the bioinformatic and biological investigation, BS exerts potentially superior protective functions on NASH livers in comparison to diet management. Furthermore, BS significantly depressed SPP1 expression, which can indicate high and poor prognosis in HCC patients. Therefore, SPP1 may serve as a novel factor to study the functionalities of BS on NASH patients.

## Data Availability

The data presented in this study are available on request from the corresponding author. The data are not publicly available due to privacy.

## Supplementary Materials

The following supporting information can be downloaded at: https://www.mdpi.com/article/10.3390/metabo13010011/s1. Figure S1: PPI network from DEGs. Figure S2: Immune cell infiltration analysis in LIHC (TCGA). Figure S3: WGCNA. Figure S4: *SPP1* in LIHC (TCGA). Figure S5: raw data batching. Table S1: Data resource.
