# Supplementary material for: Bariatric Surgery Associates with Nonalcoholic Steatohepatitis/Hepatocellular Carcinoma Amelioration via SPP1 Suppression
Source: Metabolites. 2022 Dec 21;13(1):11. doi: 10.3390/metabo13010011 (PMC9867453; doi:10.3390/metabo13010011)
Supplement: Supplementary file 1 [file metabolites-13-00011-s001.zip › metabolites-2102037-supplementary.pdf]

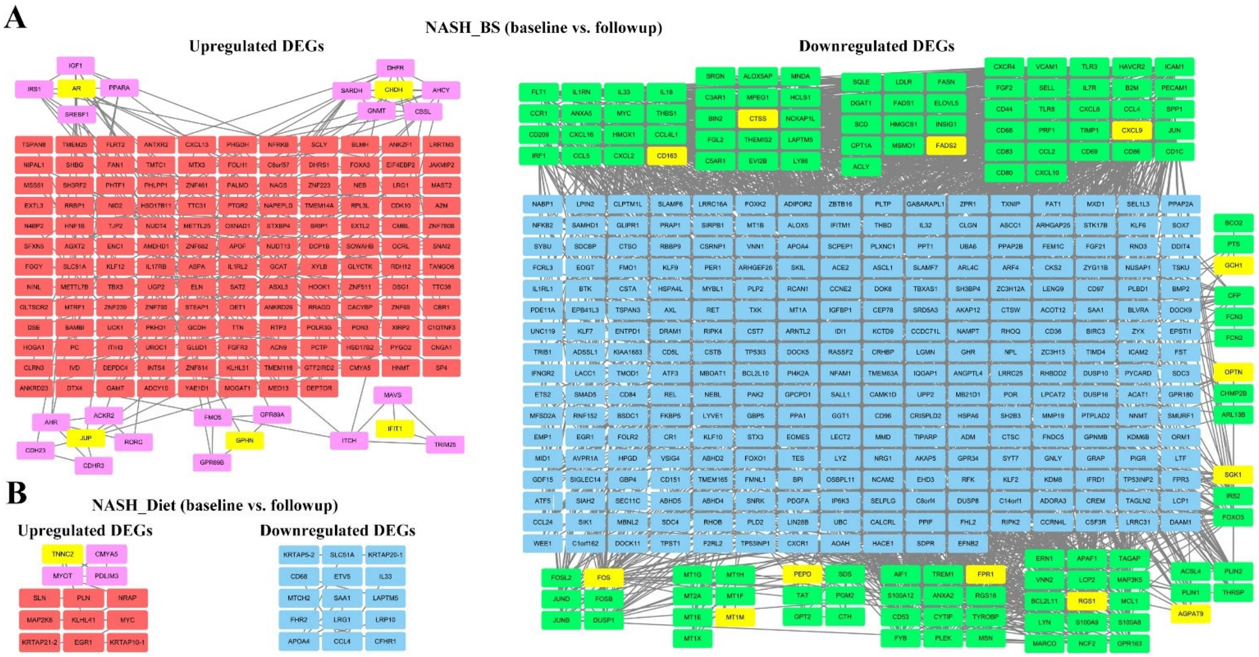

Figure S1. PPI network from DEGs. Significant upregulated and downregulated DEGs from (**A**) NASH_BS and (**B**) NASH_Diet subgroups were displayed in PPI networks. Hub gene clusters were colored (upregulated: light purple, downregulated: light green).

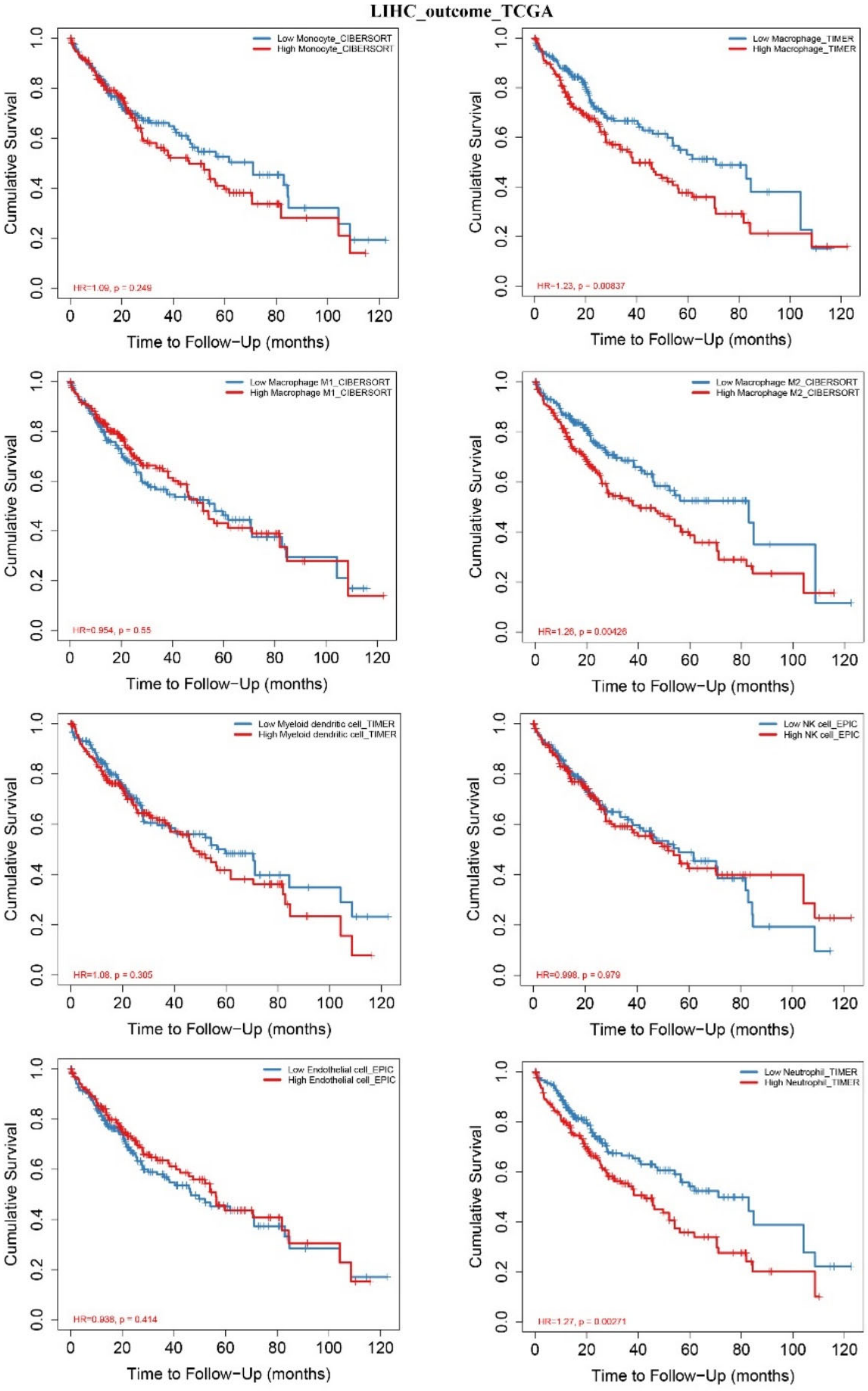

Figure S2. Immune cell infiltration analysis in LIHC (TCGA). Correlation of OS and 8 immune cell (monocyte, macrophage, M1 macrophage, M2 macrophage, DC, NK cell, endothelial cell and neutrophil) infiltration levels were analyzed in LIHC (TCGA) dataset.

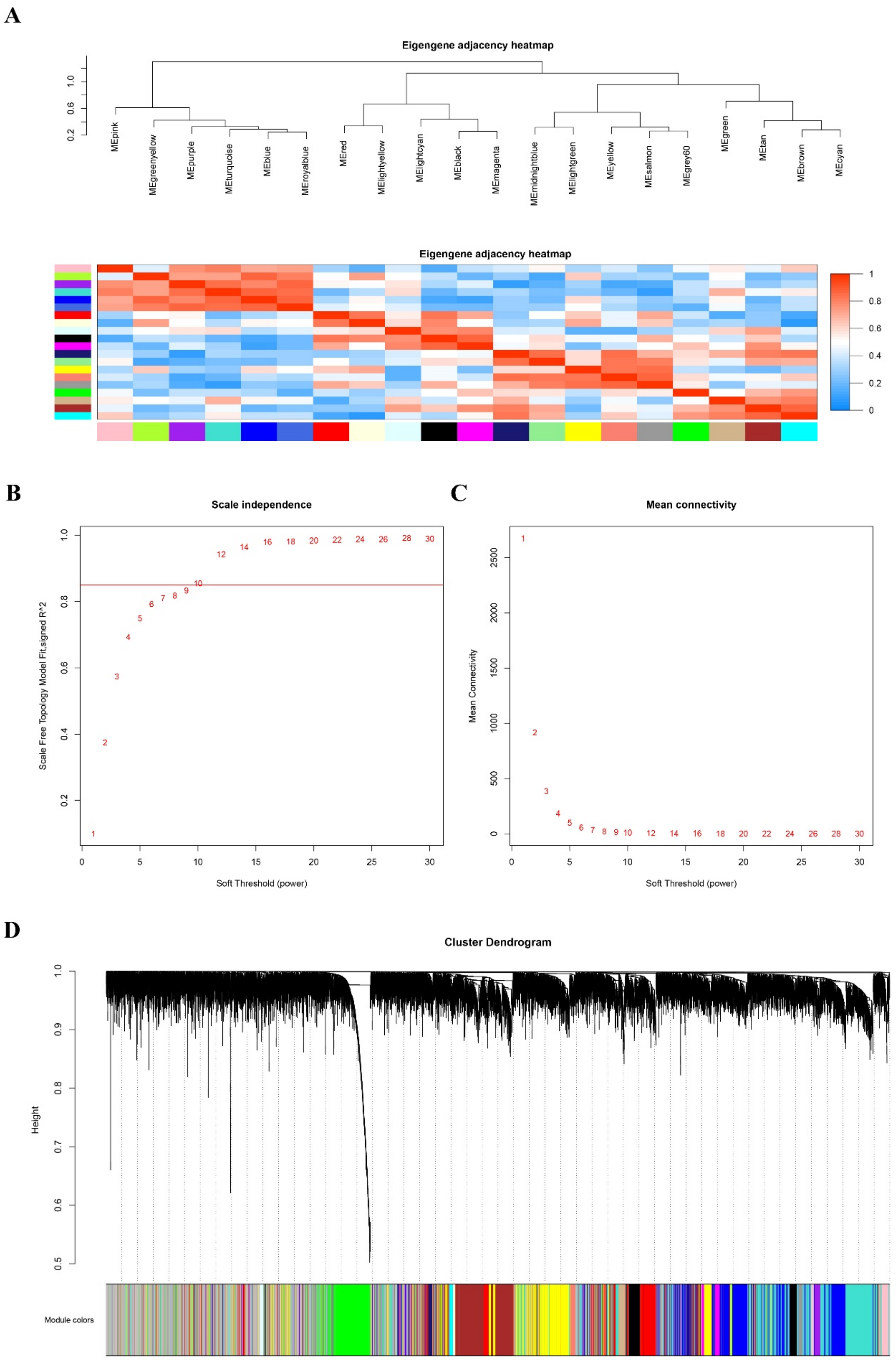

Figure S3. Within WGCNA, (**A**) Eigengene adjacency heatmap, (**B**) Scale independence, (**C**) Mean connectivity and (**D**) Cluster Dendrogram are display.

Figure S4

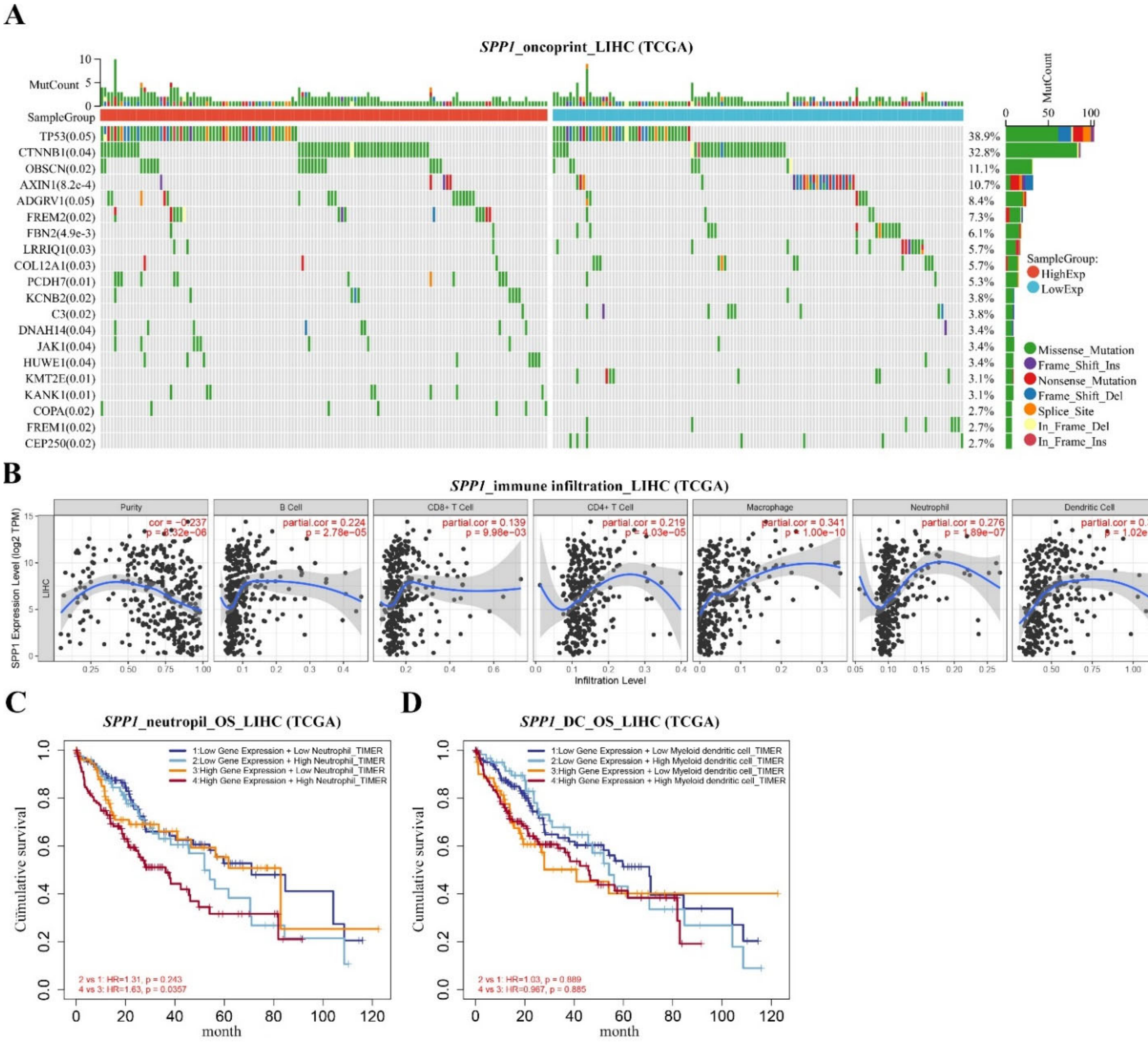

Figure S4. *SPP1* in LIHC (TCGA). (**A**) *SPP1* related gene mutation landscape was generated from LIHC (TCGA) dataset. (**B**) Correlations of *SPP1* expression and immune cell (B cell, CD8+ T cell, CD4+ T cell, macrophage, neutrophil and DC) infiltration levels were analyzed in LIHC (TCGA) dataset. Correlation of OS, *SPP1* expression and immune cell [(**C**) neutrophil and (**D**) DC] infiltration levels were analyzed in LIHC (TCGA) dataset.

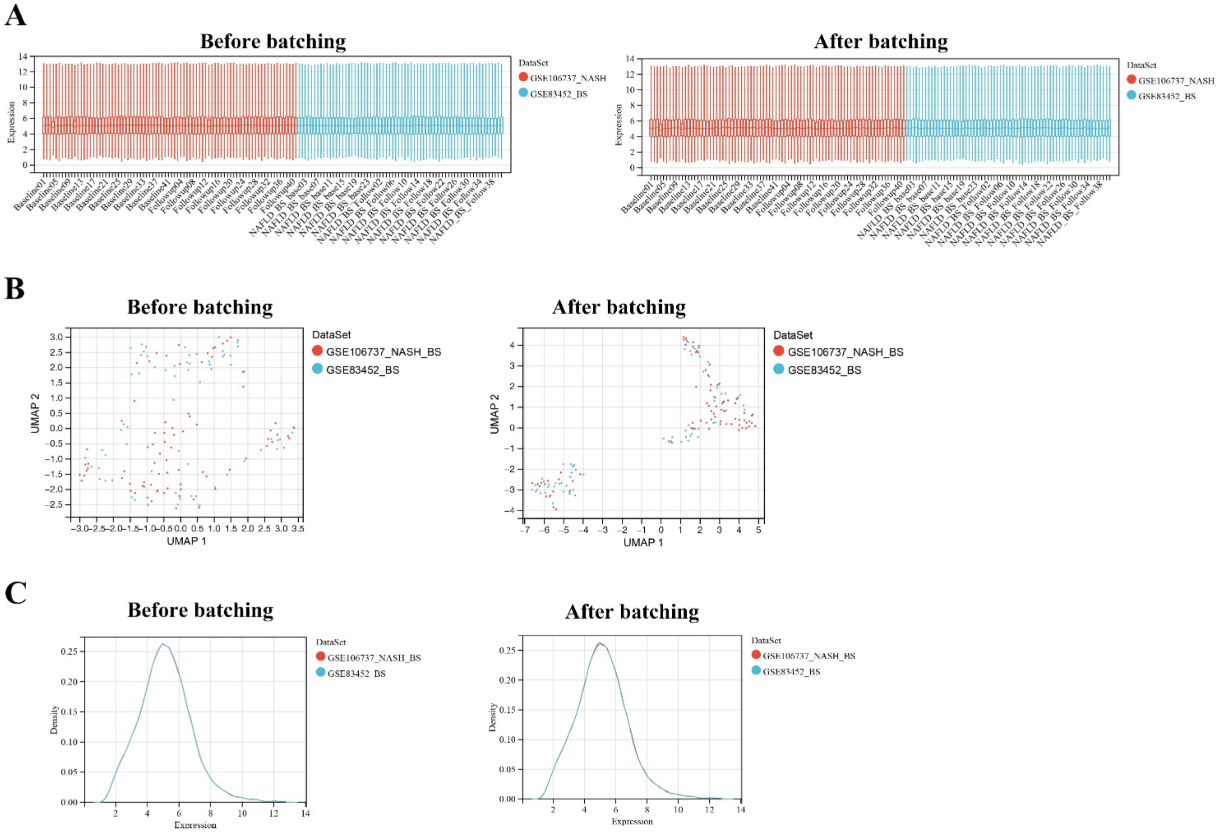

Figure S5. Raw data of GSE106737 and GSE83452 are merged. Batch effects are removed by the method of Johnson WE et al. Alterations of (**A**) gene expressions, (**B**) data densities and (**C**) UMAP are displayed as before vs. after batching.

Table S1. Data resource (bulk RNA-sequencing analysis).

ID	Samples		
	Status	Organism	Tissue
GSE83452	NASH_BS: baseline (n=16), followup (n=16)	Homo sapiens	Liver
	NASH_Diet: baseline (n=50), followup (n=20)	Homo sapiens	Liver
GSE106737	NASH_BS: baseline (n=41), followup (n=41)	Homo sapiens	Liver
GSE164760	Normal (n=6), NASH (n=74), NASH-derived HCC (n=53)	Homo sapiens	Liver
GSE89632	Normal (n=24), NASH (n=19)	Homo sapiens	Liver
GSE63067	Normal (n=7), NASH (n=9)	Homo sapiens	Liver
GSE48452	Normal (n=14), NASH (n=18)	Homo sapiens	Liver
GSE49541	Mild fibrosis (n=6), Advanced fibrosis (n=74)	Homo sapiens	Liver
